# Prevalence of stigma among patients with schizophrenia: a multi-country systematic review and meta-analysis

**DOI:** 10.3389/fpsyg.2025.1673832

**Published:** 2025-11-03

**Authors:** Xianhua Wang, Jiangli Hu, Xiaoxia Wang, Jing Liu, Yunqiong Wang

**Affiliations:** Sichuan Provincial Center for Mental Health, Sichuan Provincial People's Hospital, School of Medicine, University of Electronic Science and Technology of China, Chengdu, China

**Keywords:** schizophrenia, prevalence, stigma, meta-analysis, systematic-review

## Abstract

**Objective:**

This study aims to systematically assess the current prevalence of stigma experienced by individuals with schizophrenia and to provide evidence that can inform the development of effective intervention strategies.

**Methods:**

A systematic search was conducted in PubMed, Embase, Ovid, Web of Science, Cochrane Library, CINAHL, CNKI, Wanfang, VIP, and CBM databases to identify studies on stigma in schizophrenia patients from their inception up to November 2024. Two independent researchers performed literature screening, data extraction, and quality assessment. Meta-analysis was carried out using Stata 17.0.

**Results:**

Fourteen studies encompassing a total of 1,872 schizophrenia patients were included in the analysis. The pooled prevalence of stigma was 75.3% (95*%CI*:0.690 − 0.816). Subgroup analyses revealed the following rates: Region: Asia 79.6% (95*%CI*:0.752 − 0.841), Africa 49.9% (95*%CI*:0.460 − 0.538); Assessment tool: PDD 83.9% (95*%CI*:0.814 − 0.864), ISMI 73.9%(95*%CI*:0.665 − 0.814); Publication year: 2018-2024 year 80.8% (95*%CI*:0.737 − 0.878), 2014-2017 year 69.7%(95*%CI*:0.595 − 0.800); Age: >60 years 79.5%(95*%CI*:0.767 − 0.824), 18-60 years 74.2% (95*%CI*:0.662 − 0.821); Disease stage: Remission phase 75.8% (95*%CI*:0.679 − 0.836), Stable phase 74.1% (95*%CI*:0.635 − 0.848); Sample size: >200 cases 73.4% (95*%CI*:0.652 − 0.816), ≤ 200 cases 80.2% (95*%CI*:0.731 − 0.873). Sensitivity analysis using the leave-one-out method demonstrated stable pooled effect sizes. Egger's test (*P* = 0.08) suggested a low risk of publication bias.

**Conclusion:**

The prevalence of stigma among individuals with schizophrenia is substantial and influenced by region, assessment instruments, age, stage of illness, and sample size. The subgroup analysis indicated a higher prevalence of stigma associated with the following factors: studies conducted in Asia, the use of the PPD scale, publication after 2018, participants aged > 60 years, Sample size: ≤ 200 cases and patients in the remission stage. Future efforts should prioritize developing culturally adapted interventions specifically aimed at reducing stigma, enhancing social functioning, and improving patients' quality of life.

**Systematic review registration:**

PROSPERO, identifier: CRD42024590215.

## 1 Introduction

Schizophrenia is a severe psychiatric disorder characterized by multidimensional abnormalities in cognition, emotion, thought, volition, and behavior. Typically emerging in early adulthood, the condition demonstrates etiological complexity, heterogeneous clinical manifestations, and a chronic course (Peng et al., 2025). Systematic reviews indicate a global point prevalence of 3.72% and a lifetime prevalence of 6.00% for schizophrenia (Wang et al., 2024). Furthermore, according to Global Burden of Disease statistics (Charlson et al., 2018), schizophrenia imposes significant burdens across individual, familial, and societal levels. Despite therapeutic advances, recovery for individuals with schizophrenia continues to face multifaceted challenges, notably persistent mental illness-related stigma.

Stigma in this context manifests as a complex psychosocial phenomenon encompassing both public stigma (societal prejudice and discrimination) and self-stigma (internalization of negative stereotypes) (Tailaiti and Wang, 2022). Current evidence indicates that individuals with schizophrenia experience disproportionately elevated levels of stigma due to symptom manifestations and persistent societal prejudices (Valery and Prouteau, 2020). This dual burden triggers detrimental outcomes, including self-devaluation, social withdrawal, treatment non-adherence, and elevated suicide risk (Ociskova et al., 2023), ultimately undermining therapeutic efficacy and quality of life. Consequently, precise quantification of stigma prevalence and the development of evidence-based interventions represent critical imperatives for improving psychosocial outcomes in this population (Capar and Kavak, 2019).

The expanding research landscape on schizophrenia-related stigma reveals substantial heterogeneity in reported prevalence rates, attributable to methodological variations in study design, assessment instruments, sample characteristics, and cultural contexts. Zhang et al. have conducted systematic reviews on stigma in patients with mental illnesses, their work does not include a specific analysis of comorbidity prevalence rates in schizophrenia (Meng and Ting, 2020; Ren et al., 2025; Lu et al., 2025). This omission may obscure the distinct stigma profile associated with this particular patient population. To resolve these discrepancies and establish a comprehensive epidemiological profile, this meta-analysis systematically synthesizes existing evidence to:

(1) quantify the pooled prevalence of stigma among individuals with schizophrenia;
(2) identify potential moderators of stigma experience;
(3) provide an evidence base for optimizing clinical practice, psychological interventions, and social support systems.

## 2 Materials and methods

### 2.1 Literature search strategy

A comprehensive search was conducted across nine major databases–PubMed, Embase, Ovid, Web of Science, Cochrane Library, CINAHL, CNKI, Wanfang, VIP, and China Biology Medicine (CBM)–to identify studies examining stigma among individuals with schizophrenia. The search encompassed records from database inception through November 2024. The strategy combined Medical Subject Headings (MeSH) terms with free-text keywords using the following Boolean query: (schizophrenia OR schizophrenias OR schizophrenic disorder OR dementia praecox) AND (social stigma OR stigma). This systematic review protocol was prospectively registered with PROSPERO (Registration ID: CRD42024590215).

### 2.2 Literature inclusion and exclusion criteria

Inclusion criteria comprised:

(1) Study population: Individuals with clinically diagnosed schizophrenia per ICD-10 (International Classification of Diseases, 10th Revision; (He et al., 2024));
(2) Assessment methodology: Studies utilizing validated stigma assessment instruments;
(3) Study design: Observational studies (cohort or cross-sectional);
(4) Research focus: Studies reporting stigma prevalence rates;
(5) Outcome measures: Clear diagnostic criteria with reported stigma prevalence rates or sufficient data for rate calculation.

Exclusion criteria encompassed:

(1) Non-English or non-Chinese publications;
(2) Non-empirical literature (e.g., conference abstracts, reviews, editorials);
(3) Duplicate publications or overlapping datasets;
(4) Irretrievable full-text articles or studies with incomplete data.

### 2.3 Literature screening and data extraction

Following duplicate removal, two independent researchers conducted a systematic two-phase screening process. Initial screening involved title and abstract review. Eligible articles subsequently underwent full-text assessment to determine final inclusion. To ensure data accuracy, a standardized dual-extraction protocol was employed. Using a predesigned template, two investigators independently extracted the following variables:

First author and publication year

Country of origin

Study design

Participant characteristics

Sample size

Assessment instruments

Stigma prevalence rates.

### 2.4 Quality assessment

Two researchers independently assessed study quality using established criteria. Discrepancies were resolved by consulting a third researcher. For cross-sectional studies, quality was evaluated with the AHRQ (Agency for Healthcare Research and Quality) tool (11 items; maximum score = 11). Studies scoring 0–3, 4–7, and 8–11 were categorized as low-, moderate-, and high-quality, respectively (Ma et al., 2020). For cohort studies, the Newcastle-Ottawa Scale (NOS) was applied (8 items; maximum score = 9), with scores of 0–3, 4–6, and ≥7 indicating low, moderate, and high quality. Low-quality studies were excluded (Stang, 2010).

### 2.5 Statistical analysis

Statistical analyses were conducted using Stata 17.0. The pooled prevalence of stigma among individuals with schizophrenia was estimated with α = 0.05. Heterogeneity was quantified using the *I*^2^ statistic. If *I*^2^ <50% and *P* > 0.10 (indicating homogeneity), a fixed-effects model was applied. If *I*^2^ ≥ 50% and *P* ≤ 0.10 (suggesting significant heterogeneity), a random-effects model was used, followed by subgroup, sensitivity, and meta-regression analyses. Publication bias was assessed via Egger's test (*P* > 0.05 indicated no significant bias) (Wu et al., 2023).

## 3 Results

### 3.1 Literature screening process and results

**Figure 1:**
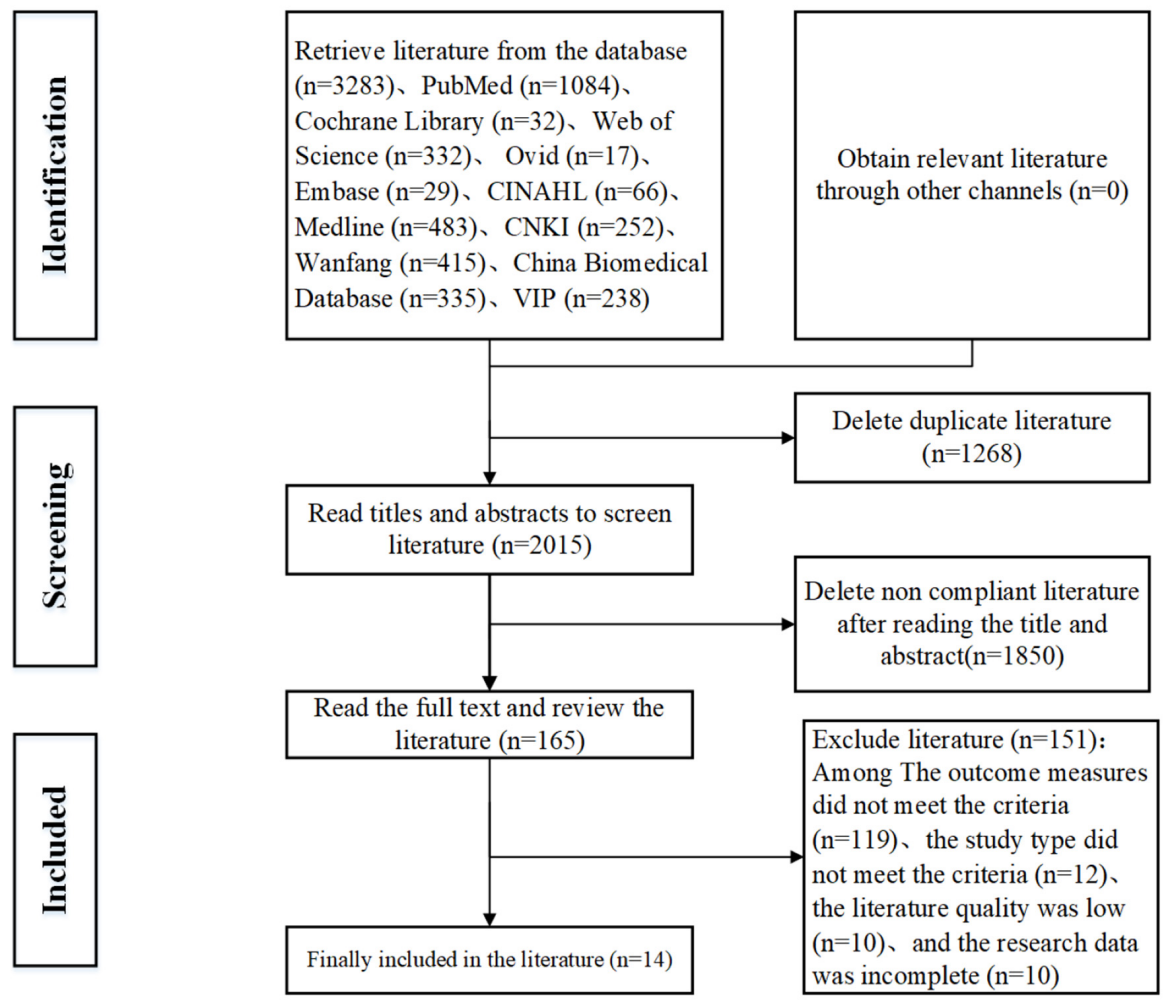
Literature screening process diagram.

A total of 3,283 records were identified through database searches. After removing 1,268 duplicates, 1,850 records were excluded during title/abstract screening. Full-text review of the remaining 165 articles excluded 151 studies failing to meet inclusion criteria, yielding 14 studies for final inclusion (Figure 1).

### 3.2 Characteristics and quality assessment of included studies

The final sample comprised 14 studies with 1,872 participants. Included designs encompassed 1 cohort study and 13 cross-sectional studies. Quality appraisal indicated high methodological quality in 8 studies (AHRQ ≥ 8 or NOS ≥ 7), as detailed in Table 1.

**Table 1:** General information and quality evaluation results of included literature.

**References**	**Area**	**Design**	**Sample size**	**Case**	**Stigma assessment tool**	**Quality**
Shi et al. (2024)	China	Cohort study	585	458	ISMI	6/9
Peng et al. (2024)	China	Cross-sectional	364	212	ISMI	8/11
Dhungana et al. (2022)	Nepal	Cross-sectional	114	102	ISMI	7/11
Pribadi et al. (2020)	Indonesia	Cross-sectional	300	243	ISMI	7/11
Tang et al. (2019)	China	Cross-sectional	425	367	PDD	8/11
Zhou et al. (2019)	China	Cross-sectional	276	248	ISMI	8/11
Deng et al. (2018)	China	Cross-sectional	215	176	ISMI	9/11
Lin et al. (2017)	China	Cross-sectional	110	88	ISMI	6/11
Yu et al. (2017)	China	Cross-sectional	272	213	ISMI	8/11
Bifftu et al. (2014)	Ethiopia	Cross-sectional	411	212	ISMI	8/11
Gao 2014)	China	Cross-sectional	152	103	PDD	7/11
Xu et al. (2014)	China	Cross-sectional	384	308	ISMI	9/11
Cao et al. (2012)	China	Cross-sectional	191	135	ISMI	8/11
Assefa et al. (2012)	Ethiopia	Cross-sectional	212	99	ISMI	7/11

PDD, Perceived Devaluation-Discrimination; ISMI, Internalized Stigma of mental illness inventory.

### 3.3 Meta-analysis of stigma prevalence in schizophrenia patients

**Figure 2:**
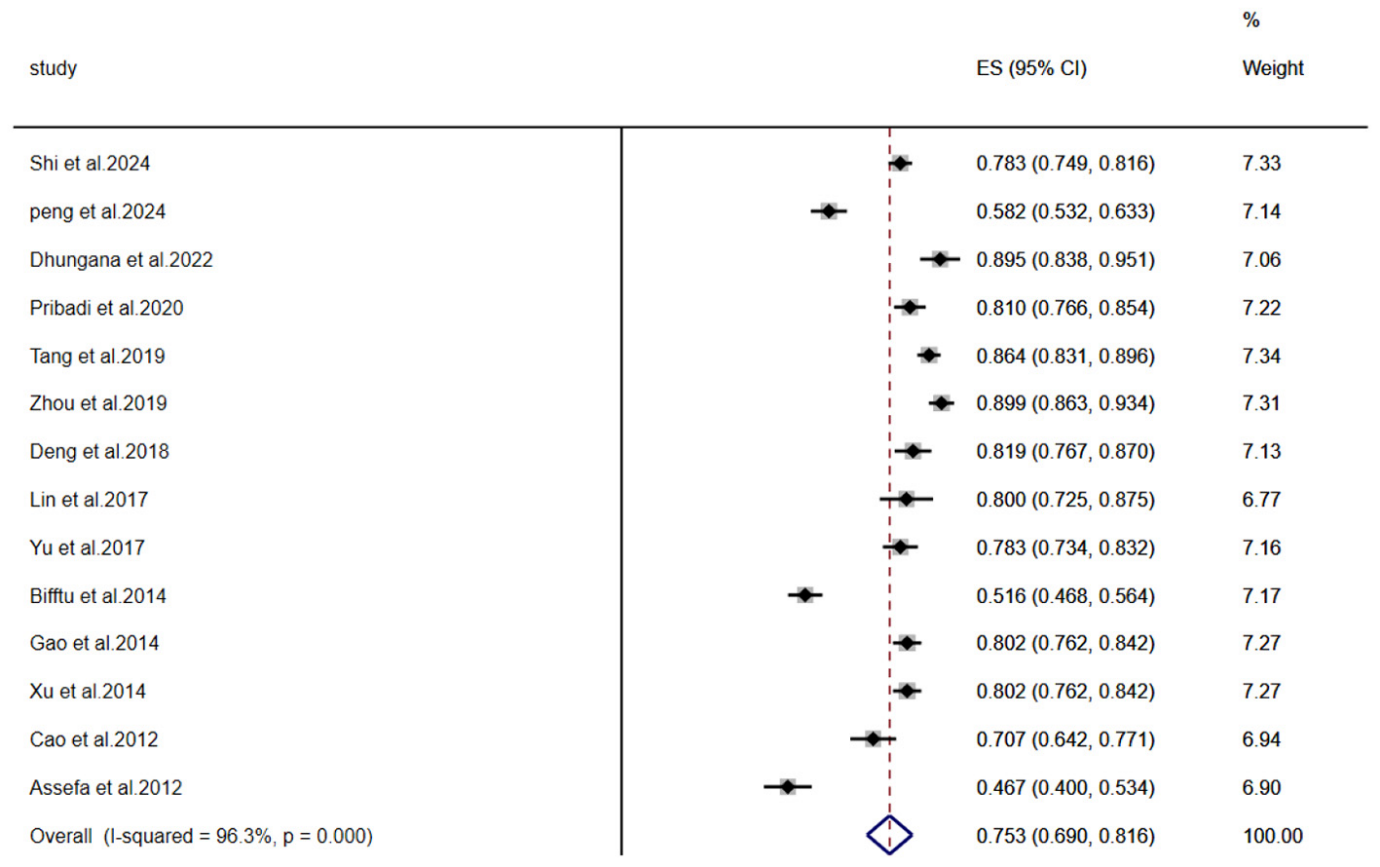
Meta-analysis of stigma prevalence in schizophrenia patients.

A meta-analysis of stigma prevalence among individuals with schizophrenia included 14 studies (*n* = 1,872). Significant heterogeneity was observed (I^2^ = 96.3%, *P* < 0.001), necessitating a random-effects model. The pooled prevalence was 75.3% (95% CI: 69.0–81.6%), as shown in Figure 2.

### 3.4 Sensitivity analysis

**Figure 3:**
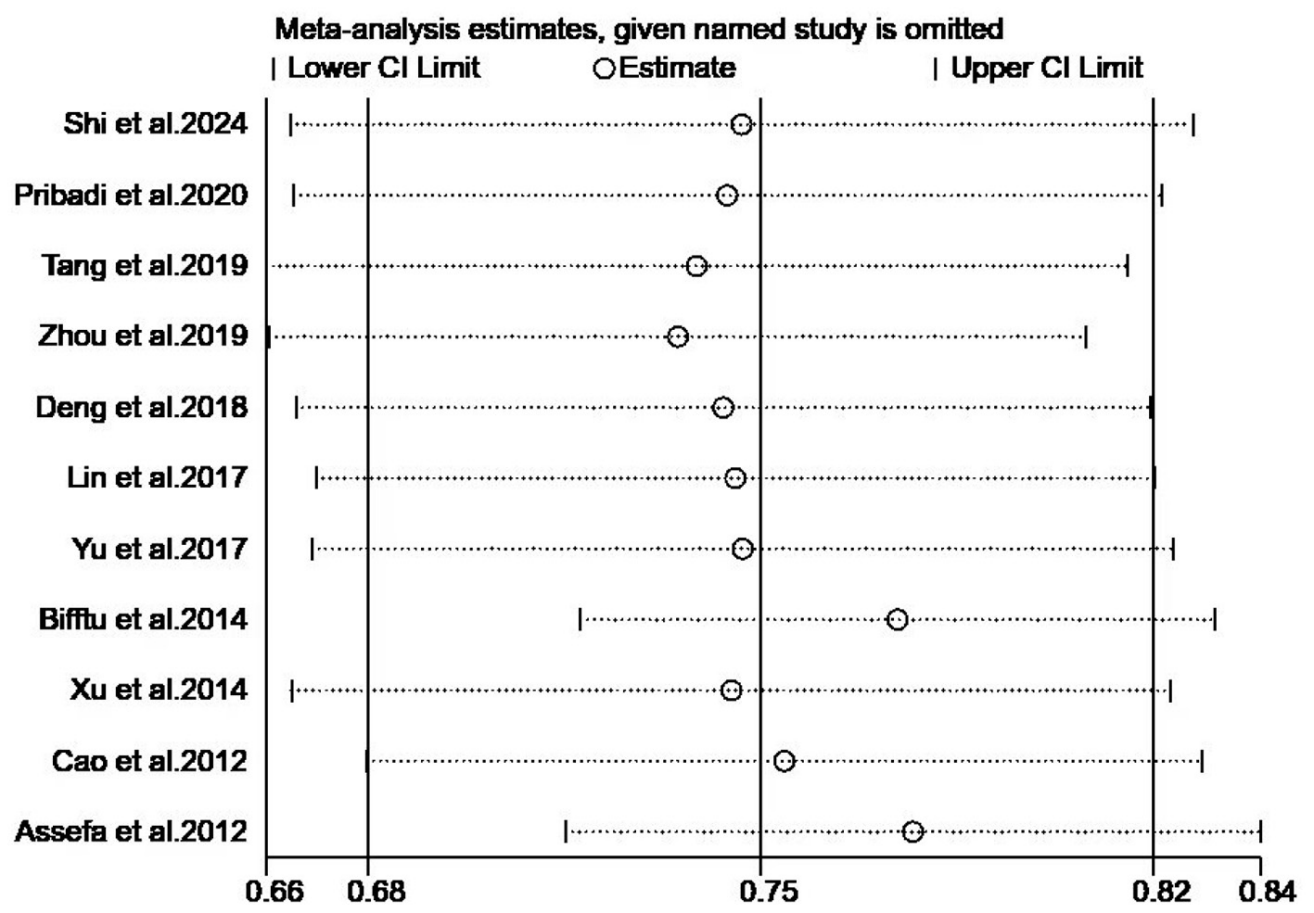
Sensitivity analysis.

Sensitivity analysis employing the leave-one-out method confirmed the stability of findings: pooled effect sizes under the random-effects model remained consistent with original estimates (Figure 3).

### 3.5 Subgroup analysis

**Figure 4:**
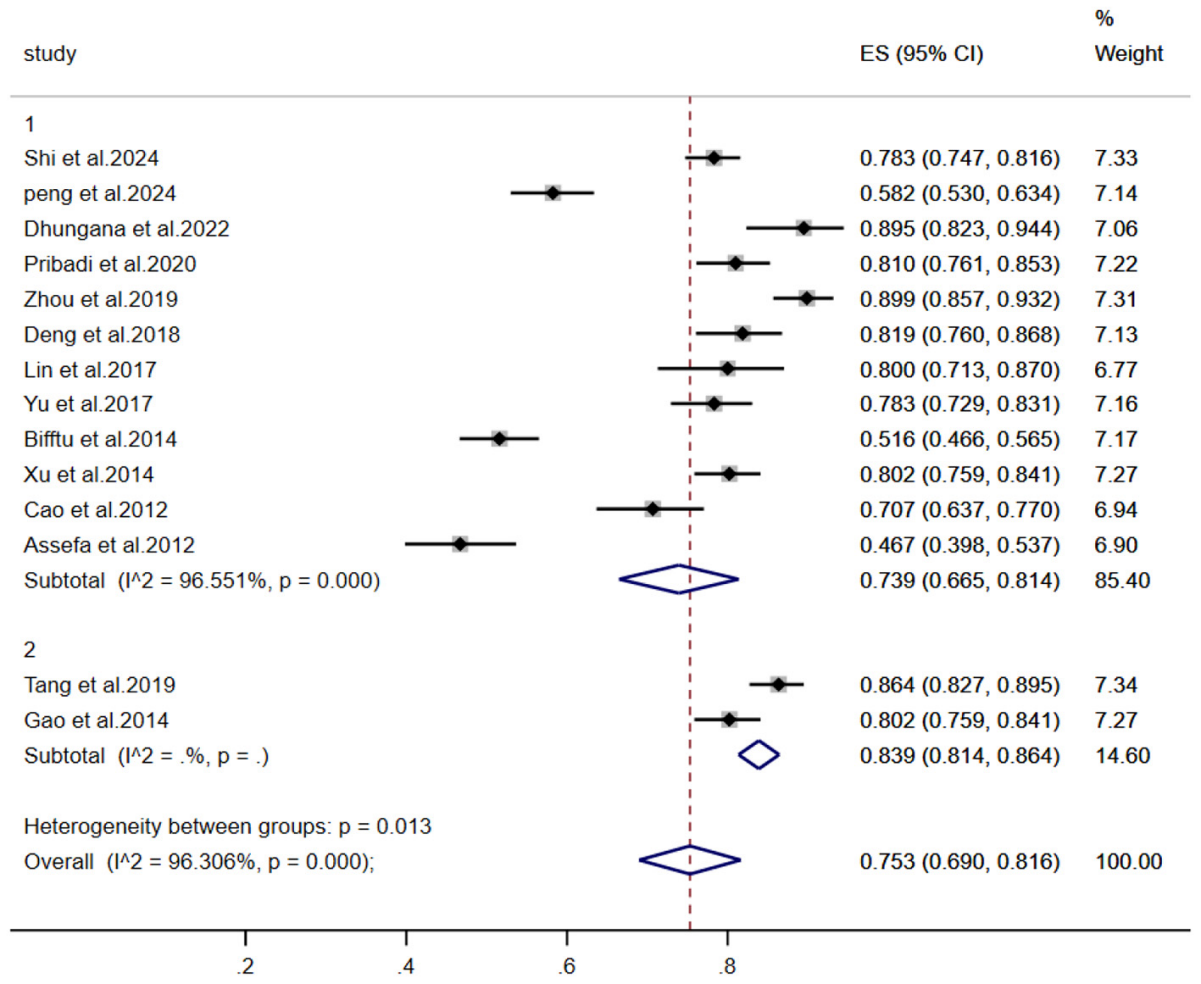
The forest plots of key subgroups (assessment tool).

**Figure 5:**
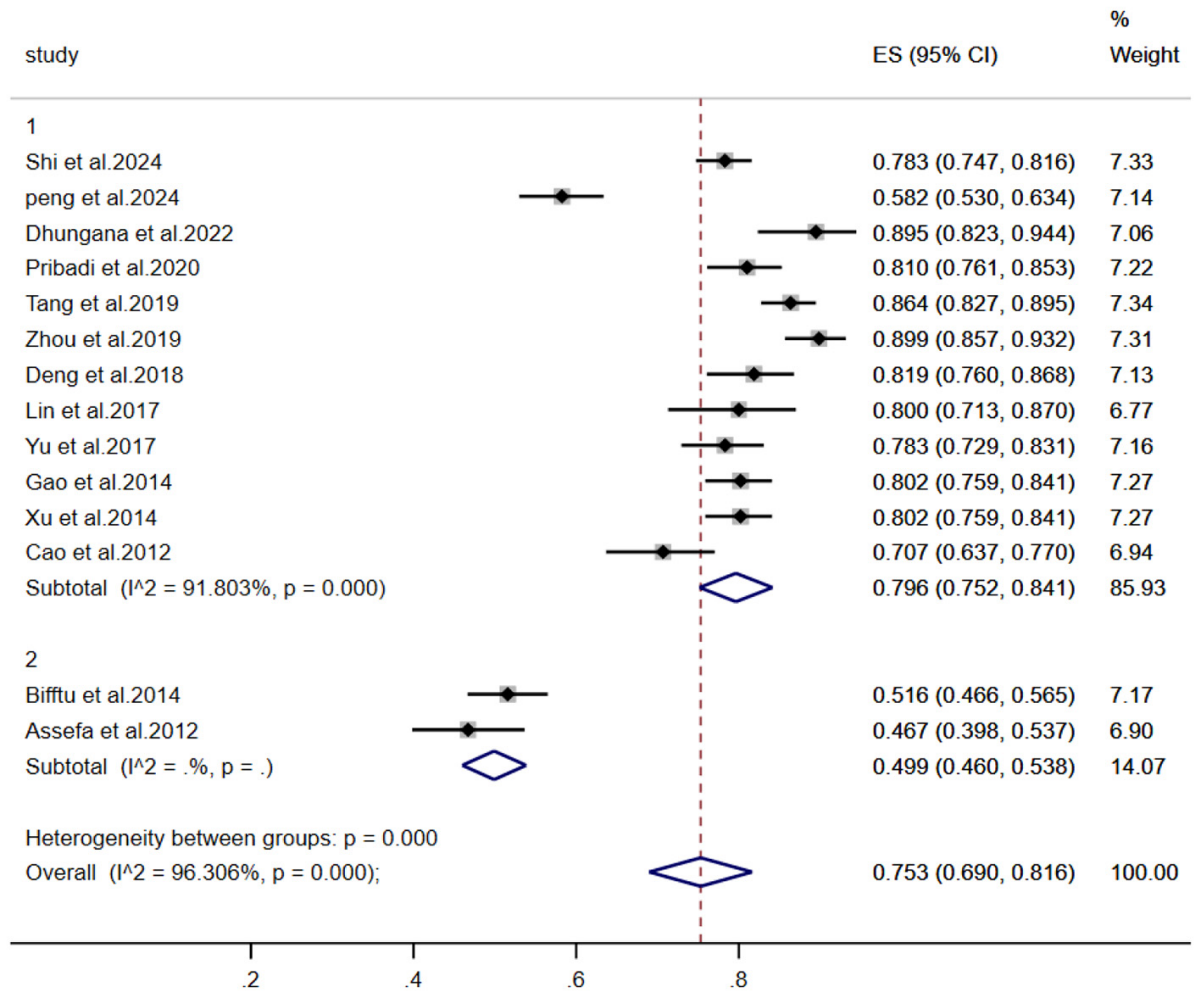
The forest plots of key subgroups (region).

To investigate heterogeneity sources, subgroup analyses utilized random-effects models across: Region of publication; ssessment instruments; Publication year; Participant age groups; Illness stage; Sample size; Comprehensive results are presented in Table 2. The forest plots of key subgroups (assessment tool) is Figure 4, the forest plots of key subgroups (region) is Figure 5.

**Table 2:** Results of subgroup analysis.

**Subgroup analysis**	**N**	**Heterogeneity**			**Meta analysis**	
		*I* ^2^	*p*	**Effect model**	**Revalence (%)**	95*%CI*
**Region**						
Ethiopia	2	/	/	Random	0.499	0.460, 0.538
Asia	12	91.80	0.00	Random	0.796	0.752, 0.841
**Assessment tool**						
PDD		/	/	Random	0.839	0.814, 0.864
ISMI	12	96.55	0.00	Random	0.739	0.665, 0.814
**Publication year**						
2012–2017	7	96.40	0.00	Random	0.697	0.595, 0.800
2018–2024	7	95.06	0.00	Random	0.808	0.737, 0.878
**Age**						
18–60	11	97.14	0.00	Random	0.742	0.662, 0.821
>60	3	/	/	Random	0.795	0.767, 0.824
**Disease stage**						
Stationary stage	4	92.21	0.00	Random	0.741	0.635, 0.848
Remission stage	10	96.89	0.00	Random	0.758	0.679, 0.836
**Sample size**						
≤ 200	4	83.92	0.00	Random	0.802	0.731, 0.873
>200	10	97.26	0.00	Random	0.734	0.652, 0.816

### 3.6 Publication bias

Egger's test assessed publication bias across the 14 studies, indicating no statistically significant bias (*P*= 0.08) and suggesting minimal publication risk.

## 4 Discussion

### 4.1 Current status of stigma in patients with schizophrenia

This study revealed a stigma prevalence of 75.3% (95% CI: 69.0–81.6%) among individuals with schizophrenia, indicating a clinically significant burden consistent with prior epidemiological reports by Shi et al. (2024) and Yu et al. (2017). To ensure diagnostic homogeneity across the included studies, we restricted eligibility to those applying the International Classification of Diseases, 10th Revision (ICD-10) criteria. While this approach may exclude studies using other diagnostic systems, such as the Diagnostic and Statistical Manual of Mental Disorders, 5th Edition (DSM-5), it strengthens the internal validity of the meta-analysis by minimizing heterogeneity and potential bias introduced by differing diagnostic standards. Pervasive societal discrimination establishes stigma as a critical psychosocial barrier during rehabilitation, substantially undermining therapeutic efficacy and social functioning (Feng et al., 2020). Current evidence identifies multidimensional determinants of stigma, including sociodemographic factors, social support availability, individual psychological characteristics (e.g., self-esteem and mastery), and clinical disease profiles (e.g., symptom severity and illness stage) (Gu and Li, 2019). As a complex phenomenon, effective stigma management necessitates integrated strategies that extend beyond symptom control to include targeted psychosocial interventions. Without timely mitigation, stigma exacerbates quality-of-life impairments, impedes social adaptation, and reduces treatment adherence, potentially accelerating disease relapse (Wang et al., 2017). Existing interventions–such as pharmacotherapy combined with cognitive behavioral therapy (CBT), self-acceptance training, and structured group psychotherapy–demonstrate variable efficacy, partly attributable to inconsistencies in intervention protocols, implementation frequency, and post-intervention follow-up durations (Zhou et al., 2024; Gao and Feng, 2023). Empirical evidence demonstrates that both contact-based and educational interventions directly reduce stigma in severe mental illness. Furthermore, family psychoeducation is associated with a significant reduction in stigma among patients (Morgan et al., 2018). Future research should prioritize multicenter large-scale randomized controlled trials (RCTs) to validate intervention scalability, optimize culturally adapted anti-stigma frameworks, and establish standardized implementation metrics, ultimately enhancing long-term patient outcomes and social inclusion.

### 4.2 Subgroup analysis

Subgroup analysis by geographic region revealed significantly higher stigma prevalence among individuals with schizophrenia in Asia (79.6%) vs. Africa (49.9%). The findings from the two Ethiopian studies are not representative of the entire Africa region. This divergence corresponds with cultural distinctions: Asian societies' emphasis on social harmony and association of mental illness with familial shame frequently lead to diagnostic concealment by patients and families, potentially exacerbating stigma internalization through reduced social support and delayed intervention (Gu and Li, 2019). The lower prevalence of stigma observed in the Africa region compared to Asia may reflect distinct cultural frameworks. In some Africa cultural contexts, the causes of mental illness may be externalized (e.g., attributed to spiritual or societal factors) rather than internalized as individual or familial failings. This externalization might buffer individuals and their families from the full negative impact of internalized stigma. Consequently, interventions aimed at reducing stigma in schizophrenia must be culturally tailored to be effective.

Subgroup analysis by assessment instrument revealed higher stigma prevalence among individuals with schizophrenia assessed with the Perceived Devaluation-Discrimination Scale (PDD; 81.4%) than with the Internalized Stigma of Mental Illness Inventory (ISMI; 66.5%), consistent with (Li 2011)'s report of elevated devaluation-discrimination perceptions. This divergence reflects the distinct stigma dimensions measured: the PDD–translated and culturally adapted for Chinese populations by (Xu 2008)—evaluates perceptions of societal devaluation and discriminatory attitudes, which may be amplified in contexts where mental illness triggers pronounced social exclusion. Conversely, the ISMI (Ritsher et al., 2003) assesses internalized stigma and affective responses (Ritsher et al., 2003), moderated by complex mediators including self-esteem, social support networks, and therapeutic engagement (Morgades-Bamba et al., 2019). Consequently, internalized stigma demonstrates lower observed prevalence due to buffering psychological mechanisms. These findings collectively underscore pervasive societal discrimination that impedes social functioning and treatment adherence, necessitating future interventions targeting cultural and psychological stigma pathways.

Subgroup analysis by publication year revealed higher stigma prevalence among individuals with schizophrenia in studies published during 2018–2024 (80.8%) vs. 2012–2017 (69.7%). This apparent increase may reflect methodological advances–including standardized assessment protocols and diagnostic refinements–enhancing stigma detection sensitivity. Paradoxically, longitudinal research by Fan et al. (2023) demonstrates improved societal awareness and reduced perceived discrimination over two decades, particularly among younger, educated patients with shorter inpatient histories. However, persistent stigma effects in older, less-educated individuals and those with extended hospitalizations suggest uneven progress. Furthermore, long-term hospitalization may foster patient dependence and a decline in social skills, thereby intensifying internalized stigma. It is also notable that despite rapid economic and educational progress, individuals who are older, have lower educational attainment, or have experienced prolonged hospitalization remain particularly vulnerable. Greater public health discourse may have simultaneously raised patient awareness of discrimination while inadequately mitigating structural barriers. Consequently, enhancing societal support systems remains critical to improving therapeutic engagement, social functioning, and long-term recovery trajectories.

Subgroup analysis by age revealed significantly higher stigma prevalence among individuals with schizophrenia aged >60 years (79.5%) vs. those aged 18–60 years (74.2%). Older patients frequently experience comorbid physical conditions, progressive functional decline in self-care and cognition (Stroup et al., 2021), and reduced social support networks (Gao, 2024). These factors collectively heighten vulnerability to illness concealment and self-stigmatization—particularly through barriers to adapting to contemporary treatment models–exacerbating helplessness and social exclusion. During this stage, most elderly individuals are mostly in retirement. They tend to perceive a loss of self-worth and have relatively poor psychological resilience. Close and supportive family environments help reduce these patients' stigma; therefore, when implementing interventions for patients, it is necessary to incorporate family care into these interventions. Within China, socio-cultural norms appear to shape stigma in distinct ways. Traditional familism and the associated concept of “face” can exacerbate both public and self-stigma. Consequently, this analysis contributes to a theoretical framework for understanding how cultural factors can exacerbate stigma in schizophrenia. Future interventions should implement geriatric-focused psychosocial strategies that address concealment behaviors, strengthen self-efficacy, and mitigate stigma-related functional impairment.

Subgroup analysis by illness stage demonstrated marginally higher stigma prevalence among individuals with schizophrenia during remission (75.8%) vs. stable phases (74.1%). This aligns with WonPat-Borja et al. (2012)'s findings of sustained discrimination across illness trajectories, with maximal self-perceived stigma occurring in remission. The pattern likely reflects dual psychological burdens: patients manage residual symptoms while navigating societal discrimination and reintegration pressures during functional recovery.

Subgroup analysis by sample size revealed significantly higher stigma prevalence in studies with ≤ 200 participants (80.2%) vs. those with >200 participants (73.4%). This divergence likely reflects methodological limitations: smaller samples are vulnerable to selection bias (e.g., overrepresentation of clinical populations with higher stigma), whereas larger samples enhance generalizability through broader demographic representation and reduced sampling error (Chen et al., 2024). The lower prevalence in larger studies may more accurately reflect population-level stigma dynamics. Future research should prioritize multicenter collaborations to achieve sufficient statistical power and employ longitudinal designs with time-lagged assessments to examine stigma trajectories and social functioning outcomes.

Limitations include: (1) Due to the predominance of cross-sectional designs, the ability to establish causal relationships is limited. Additionally, the diversity of assessment tools and the limited number of studies restrict the generalizability of the findings; (1) predominance of cross-sectional designs, constraining causal inference; (2) significant heterogeneity in this single-arm meta-analysis, attributable to methodological and cultural variations; (3) limited subgroup statistical power and unresolved heterogeneity sources affecting precision. (4) restriction to English and Chinese publications, which may introduce language and geographic bias.

## 5 Conclusion

In summary, this meta-analysis confirms a persistently high prevalence of stigma among individuals with schizophrenia. Healthcare systems should implement geriatric-focused protocols for older patients and targeted interventions for those in remission, while standardizing assessment instruments using culturally validated metrics. Evidence indicates that community- and family-based interventions can effectively mitigate stigma while also improving clinical symptoms and social functioning in patients with schizophrenia. Moving forward, the management of this disorder should transition toward an integrated model that synergizes community screening, long-term management, and public education. Establishing evidence-based anti-stigma frameworks—integrating pharmacological, psychosocial, and community-based approaches—is critical for enhancing functional recovery and quality of life. The digital health tools represent a promising avenue for addressing stigma in the future. A key direction for subsequent research will be to specifically explore the role of AI-driven digital platforms in stigma intervention and support (Lee et al., 2024). Machine learning offers a promising avenue for developing predictive models of mental health outcomes in individuals with schizophrenia. Such models could identify complex, multivariate predictors of stigma—including emotional intelligence and social skills—thereby providing a evidence-based foundation for formulating personalized intervention strategies (Tuan et al., 2024; Ramos-Galarza et al., 2024). Future research must investigate intervention efficacy through multicenter randomized trials and establish cultural adaptation frameworks to ensure global applicability of stigma-reduction strategies.

## Data Availability

The original contributions presented in the study are included in the article/supplementary material, further inquiries can be directed to the corresponding author.
